# On the Origin of the Vaccine Inoculation

**Published:** 1801-06

**Authors:** Edward Jenner

**Affiliations:** Bond Street


### *On the Origin of the Vaccine Inoculation.*

THE moft important difcoveries, when familiarized to the mind, are contemplated with indifference. Who now wonders at the difcovery of America, or the circulation of the blood? There is, however, a period between the conception of a difcovery and its mature birth, fraught with more pangs than war or women know; and there is no light, in which the human mind can be viewed, more interefting than during this anxious period. Whenever, therefore, the author of any greatly ufeful invention details the progrefs of his own mind, during the completion of his plan, the hiftory is perufed with avidity. On thefe grounds, we conclude that our readers will be much gratified by the following narrative.

" *I am induced to give the following concise History of the Origin of Vaccine Inoculation, from my frequently observing that those who only consider the subject cursorily, confound the casual Cow Pox with the Disease when excited by Inoculation.*

*Bond Street, May* 6, 1801. *EDWARD JENNER.*

" My inquiry into the nature of the Cow-pox commenced upwards of twenty-five years ago. My attention to this fingular difeafe was firft excited by obferving, that among thofe whom in the country I was frequently called upon to inoculate, many refifted every effort to give them the Small-pox. Thefe patients I found had undergone a difeafe they called the Cow-pox, contracted by milking Cows affected with a peculiar eruption on their teats. On inquiry, it appeared that it had been known among the dairies time immemorial, and that a vague opinion prevailed that it was a preventive of the Small Pox. This opinion I found was, comparatively, new among them; for all the older farmers declared they had no fuch idea in their early days—a circumftance that feemed eafily to be accounted for, from my knowing that the common people were very rarely inoculated for the Small-pox, till that practice was rendered general by the improved method introduced by the Suttons: fo that the working people in the dairies were feldom put to the teft of the preventive powers of the Cow-pox.

"In the courfe of the inveftigation of this fubject, which, like all others of a complex and intricate nature, prefented many difficulties, I found that fome of thofe *who feemed to have undergone the Cow-pox*, neverthelefs, on inoculation with the Small-pox, felt its influence juft the fame as if no difeafe had been communicated to them from the cow. This occurrence led me to enquire among the medical practitioners in the country around me, who all agreed in this fentiment, that the Cow-pox was not to be relied upon as a certain preventive of the Small-pox. This for a while damped but did not extinguifh my ardour; for as I proceeded, I had the fatisfaction to learn that the cow was fubject to fome varieties of fpontaneous eruptions upon her teats; that they were all capable of communicating fores to the hands of the milkers; and that whatever fore was derived from the animal, was called in the dairy the Cow-pox. Thus I furmounted a great obftacle, and, in confequence, was led to form a diftinction between thefe difeafes, one of which only I have denominated the *true*, the others the *fpurious*, Cow-pox, as they poffefs no fpecific power over the conftitution. This impediment to my progrefs was not long removed, before another, of far greater magnitude in its appearances, ftarted up. There were not wanting inftances to prove, that when the true Cow-pox broke out among the cattle at a dairy, a perfon who had milked an infected animal, and had thereby apparently gone through the difeafe in common with others, was liable to receive the Small-pox afterwards. This, like the former obftacle, gave a painful check to my fond and afpiring hopes: but reflecting that the operations of Nature are generally uniform, and that it was not probable the human conftitution (having undergone the Cow-pox) fhould in fome inftances be perfectly fhielded from the Small-pox, and in many others remain unprotected, I refumed my labours with redoubled ardour. The refult was fortunate; for I now difcovered that the virus of Cow-pox was liable to undergo progreffive changes, from the fame caufes precifely as that of Small-pox; and that when it was applied to the human fkin in its degenerated ftate, it would produce the ulcerative effects in as great a degree as when it was not not decompofed, and fometimes far greater; but having loft *its fpecific properties*, it was incapable of producing that change upon the human frame which is requifite to render it unfufceptible of the variolous contagion: fo that it became evident a perfon might milk a cow one day, and having caught the difeafe, be for ever fecure; while another perfon, milking the fame cow the next day, might feel the influence of the virus in fuch a way, as to produce a fore or fores, and in confequence of this might experience an indifpofition to a confiderable extent; yet, as has been obferved, the fpecific quality being loft, the conftitution would receive no peculiar impreffion.

"Here the clofe analogy betwen the Virus of Small-pox and of Cow-pox becomes remarkably confpicuous; fince the former, when taken from a recent puftule, and immediately ufed, gives the perfect Small-pox to the perfon on whom it is inoculated: but when taken in a far advanced ftage of the difeafe, or when (although taken early) previoufly to its infertion, it be expofed to fuch agents as, according to the eftablifhed laws of Nature, caufe its decompofition, it can no longer be relied on as effectual. This obfervation will fully explain the fource of thofe errors which have been committed by many inoculators of the Cow-pox. Conceiving the whole procefs to be fo extremely fimple, as not to admit of a miftake, they have been heedlefs about the ftate of the Vaccine Virus; and finding it limpid, as part of it will be, even in an advanced ftage of the puftule, when the greater portion has been converted into a fcab, they have felt an improper confidence, and fometimes miftaken a fpurious puftule, which the Vaccine fluid in this ftate is capable of exciting, for that which poffeffes the perfect character.

"During the inveftigation of the cafual Cow-pox, I was ftruck with the idea that it might be practicable to propagate the difeafe by inocoulation, after the manner of the Small-pox, firft from the Cow, and finally from one human being to another. I anxioufly waited fome time for an opportunity of putting this theory to the teft. At length the period arrived. The firft experiment was made upon a lad of the name of Phipps, in whofe arm a little Vaccine Virus was inferted, taken from the hand of a young woman who had been accidentally infected by a cow. Notwithftanding the refemblance which the puftule, thus excited on the boy's arm, bore to variolous inoculation, yet as the indifpofition attending it was barely perceptible, I could fcarcely perfuade myfelf the patient was fecure from the Small-pox. However, on his being inoculated fome months afterwards, it proved that he was fecure. This cafe infpired me with confidence; and as foon as I could again furnifh myfelf with virus from the Cow, I made an arrangement ment for a ſeries of inoculations. A number of children were inoculated in ſucceſſion, one from the other; and after ſeveral months had elapſed, they were expoſed to the infection of the Small-pox; ſome by inoculation, others by variolous effluvia, and ſome in both ways; but they all reſiſted it. The reſult of theſe trials gradually led me into a wider field of experiment, which I went over not only with great attention but with painful ſolicitude. This became univerſally known through a Treatiſe publiſhed in June 1798. The reſult of my further experience was alſo brought forward in ſubſequent publications in the two ſucceeding years, 1799 and 1800. The diſtruſt and ſcepticiſm which naturally aroſe in the minds of medical men, on my firſt announcing ſo unexpected a diſcovery, has now nearly diſappeared. Many hundreds of them, from actual experience, have given their atteſtations that the inoculated Cow Pox proves a perfect ſecurity againſt the Small Pox; and I ſhall probably be within compaſs if I ſay, thouſands are ready to follow their example; for the ſcope that this inoculation has now taken is immenſe. An hundred thouſand perſons, upon the ſmalleſt computation, have been inoculated in theſe realms. The numbers who have partaken of its benefits throughout Europe, and other parts of the globe, are incalculable; and it now becomes too manifeſt to admit of controverſy, that the annihilation of the Small-pox, the moſt dreadful ſcourge of the human ſpecies, muſt be the final reſult of this practice.

